# Plasma exchange successfully treats central pontine myelinolysis after acute hypernatremia from intravenous sodium bicarbonate therapy

**DOI:** 10.1186/1471-2369-15-56

**Published:** 2014-04-04

**Authors:** Kyung Yoon Chang, In-Hee Lee, Gi Jun Kim, Kangwon Cho, Hoon Suk Park, Hyung Wook Kim

**Affiliations:** 1 Division of Nephrology, Department of Internal Medicine, St. Vincent’s Hospital, The Catholic University of Korea, Seoul, Korea

**Keywords:** Central pontine myelinolysis, Hypernatremia, Plasma exchange, Renal tubular acidosis, Sjögren’s syndrome, Sodium bicarbonate

## Abstract

**Background:**

Osmotic demyelination syndrome (ODS) primarily occurs after rapid correction of severe hyponatremia. There are no proven effective therapies for ODS, but we describe the first case showing the successful treatment of central pontine myelinolysis (CPM) by plasma exchange, which occurred after rapid development of hypernatremia from intravenous sodium bicarbonate therapy.

**Case presentation:**

A 40-year-old woman presented with general weakness, hypokalemia, and metabolic acidosis. The patient was treated with oral and intravenous potassium chloride, along with intravenous sodium bicarbonate. Although her bicarbonate deficit was 365 mEq, we treated her with an overdose of intravenous sodium bicarbonate, 480 mEq for 24 hours, due to the severity of her acidemia and her altered mental status. The next day, she developed hypernatremia with serum sodium levels rising from 142.8 mEq/L to 172.8 mEq/L. Six days after developing hypernatremia, she exhibited tetraparesis, drooling, difficulty swallowing, and dysarthria, and a brain MRI revealed high signal intensity in the central pons with sparing of the peripheral portion, suggesting CPM. We diagnosed her with CPM associated with the rapid development of hypernatremia after intravenous sodium bicarbonate therapy and treated her with plasma exchange. After two consecutive plasma exchange sessions, her neurologic symptoms were markedly improved except for mild diplopia. After the plasma exchange sessions, we examined the patient to determine the reason for her symptoms upon presentation to the hospital. She had normal anion gap metabolic acidosis, low blood bicarbonate levels, a urine pH of 6.5, and a calyceal stone in her left kidney. We performed a sodium bicarbonate loading test and diagnosed distal renal tubular acidosis (RTA). We also found that she had Sjögren’s syndrome after a positive screen for anti-Lo, anti-Ra, and after the results of Schirmer’s test and a lower lip biopsy. She was discharged and treated as an outpatient with oral sodium bicarbonate and potassium chloride.

**Conclusion:**

This case indicates that serum sodium concentrations should be carefully monitored in patients with distal RTA receiving intravenous sodium bicarbonate therapy. We should keep in mind that acute hypernatremia and CPM can be associated with intravenous sodium bicarbonate therapy, and that CPM due to acute hypernatremia may be effectively treated with plasma exchange.

## Background

Osmotic demyelination syndrome (ODS), which is also known as central pontine myelinolysis (CPM) or extra-pontine myelinolysis, primarily occurs after the rapid correction of severe hyponatremia. The clinical manifestations of ODS are typically delayed for two to six days after swift elevations in the serum sodium level. The symptoms, which are often irreversible or only partially reversible, include dysarthria, dysphagia, tetraparesis, behavioral disturbances, lethargy, confusion, disorientation, and coma [1]. ODS can also occur during other osmotic challenges such as rapid correction of hypernatremia [2] and the development of acute hypernatremia from normal sodium concentrations [3,4].

When CPM is associated with the rapid correction of hyponatremia, plasma exchange (plasmapheresis) can be a beneficial therapy. In fact, Bibl et al. reported that three young female patients with CPM, which associated with correction of hyponatremia, were successfully treated with extensive therapeutic plasma exchange [5]. However, ours is the first case report of CPM after rapid development of hypernatremia from sodium bicarbonate therapy to be successfully treated by plasma exchange.

## Case presentation

A 40-year-old woman presented with general weakness, nausea, vomiting, numbness, and weight loss of 8 kg over the previous two months. The patient was 154 cm tall and weighed 57 kg. Her blood pressure was 100/60 mmHg and pulse was 110 beats/min. She was on no diuretics or other medications. She did not have a medical history or diarrhea upon presentation. On admission, she had weakness in both limbs (Medical Research Council Grade 2), but her deep-tendon reflexes were intact. Babinski’s sign and ankle clonus were absent.

Upon initial laboratory analysis, her sodium was 142.8 mEq/L; potassium, 2.3 mEq/L; chloride, 125.5 mEq/L; calcium, 7.7 mg/dL; phosphorus, 1.1 mg/dL; magnesium, 2.6 mg/dL; blood urea nitrogen, 17.7 mg/dL; creatinine, 1.0 mg/dL; and albumin, 4.2 g/dL. Her spot urine potassium was 16.9 mEq/L, and the transtubular potassium gradient (TTKG) was 7%, suggesting renal loss. Blood gas analysis revealed a pH of 7.194, PCO_2_ of 19.5 mmHg, PO_2_ of 67.8 mmHg, HCO_3_ of 7.6 mEq/L, SpO_2_ of 90.2%, and a serum anion gap of 9.7, suggesting normal anion gap metabolic acidosis. Urinalysis revealed a pH of 6.5 and a urine anion gap of 6.1.

Due to the patient’s hypotension and altered mental status, she was admitted to the intensive care unit. She required mechanical ventilatory support with supplemental oxygen due to lethargy, somnolence, and respiratory failure (PCO_2_ 45.3 mmHg). She was treated with intravenous potassium chloride and an oral potassium chloride tablet via nasogastric tube.

**Figure 1:**
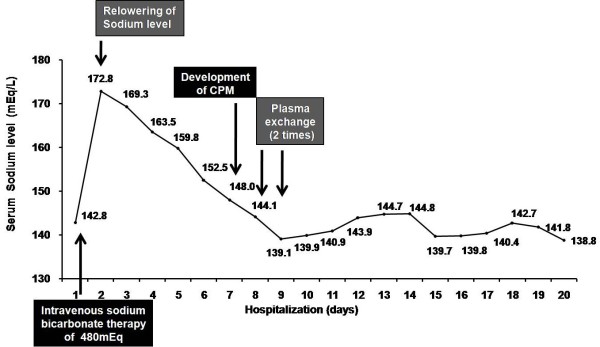
Sodium level changes.

In addition to ventilatory support, she also required treatment for her severe acidemia with sodium bicarbonate. Her desired bicarbonate level was 24 mEq, and her bicarbonate deficit was calculated to be 365 mEq from the following formula: bicarbonate deficit = (0.5 × lean body weight) × (24- serum bicarbonate) [6]. Her lean body weight was calculated to be 44.5 kg from the following formula: lean body weight [kg] = 9270 × body weight [kg]/6680 + (216 x BMI [kg/m^2^]). We treated her with an overdose of 480 mEq of intravenous sodium bicarbonate in 5% dextrose and water solution because her mental status and severe metabolic acidosis did not improve. The next day, her potassium was corrected to normal levels (4.2 mEq/L). However, we found acute hypernatremia with her sodium levels rising from 142.8 mEq/L to 172.8 mEq/L. We began treating her with half-normal saline and 5% dextrose in water, and her serum sodium level gradually decreased from 172.8 mEq/L to 148.0 mEq/L over the course of six days (Figure 1). As her hypernatremia was corrected, she became more alert and her weakness was greatly improved.

**Figure 2:**
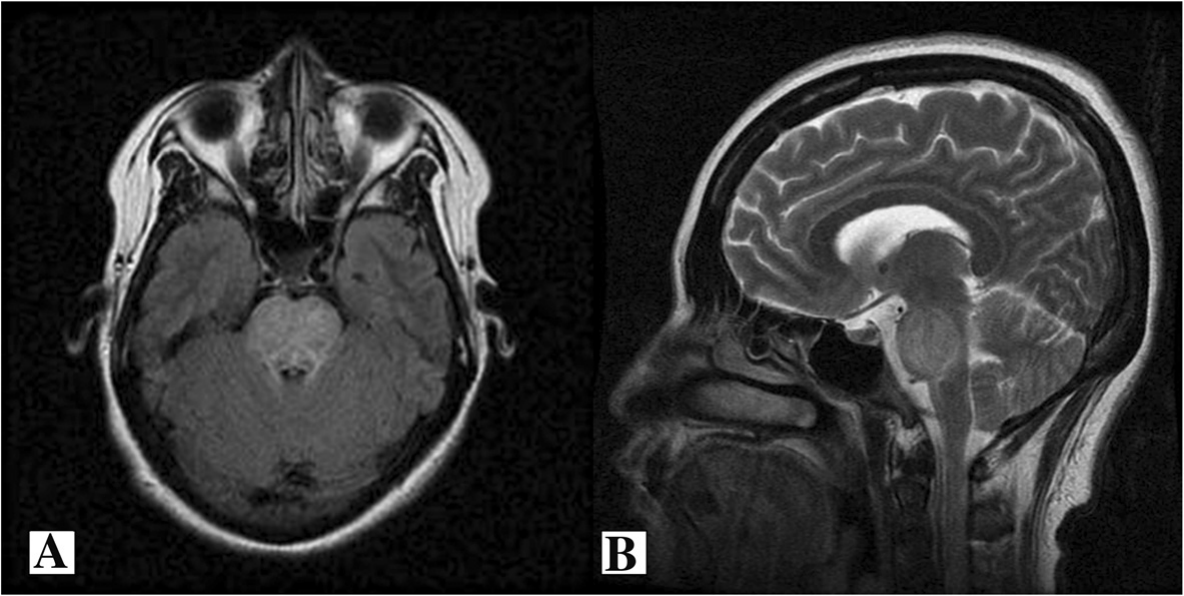
MRI of the brain revealed symmetric, high-intensity signal in the pons with sparing of the peripheral portion, suggesting central pontine myelinolysis (A, axial view and B, sagittal view).

Six days after the development of hypernatremia, several other symptoms became evident including dysarthria, drooling, difficulty swallowing, and tetraparesis. Because of these symptoms, we suspected ODS associated with acute hypernatremia. Consistent with our clinical suspicion, a brain MRI revealed symmetric, high-intensity signal in the central pons with sparing of the peripheral portion, suggesting CPM (Figure 2). Soon after this diagnostic confirmation of CPM, two consecutive therapeutic plasma exchange sessions for two days were started with a total of 4394 mL plasma exchanged with albumin 5%, crystalloids, and fresh frozen plasma. The day following the plasma exchange, she regained speech and became oriented. Her neurological symptoms, which included dysarthria, difficulty swallowing, and tetraparesis, were markedly improved, but mild diplopia was present.

After the patient was stabilized, we were able to work her up to explore the primary cause of her presenting symptoms. We suspected distal renal tubular acidosis (RTA) due to the normal anion gap metabolic acidosis, a serum bicarbonate level < 10, hypokalemia, and urine pH of 6.5 (> 5.5), and due to the presence of a calyceal stone in her left kidney. We performed a sodium bicarbonate (NaHCO_3_) loading test to confirm distal RTA [7]. In NaHCO_3_ loading test, 8.4% NaHCO_3_ solution was infused intravenously at a rate of 57 mEq/hour (1 mEq/kg/hour). Urine and blood samples were taken at 1-hour intervals and urine and blood PCO_2_ were measured using a blood gas analyzer. When the urine pH was raised to 7.6, urine PCO_2_, blood PCO_2_, urine HCO_3_, blood HCO_3_, urine creatinine and serum creatinine were 44.5 mmHg, 34.8 mmHg, 40.1 mEq/L, 25.8 mEq/L, 64.5 mg/dL, and 0.7 mg/dL, respectively. The results of NaHCO_3_ loading test revealed a fractionated excretion of HCO_3_ of 1.68% and the urine-to-blood carbon dioxide tension gradient (U-B PCO_2_) of 9.7, suggesting distal RTA. In addition to the distal RTA, we also found that she had Sjögren’s syndrome after the results of Schirmer’s test and after she screened positive for anti-Lo and anti-Ra, which was confirmed by a salivary scan and lower lip biopsy. She was discharged and treated at an outpatient clinic with oral sodium bicarbonate and potassium chloride. After one year of follow-up, her neurological symptoms were improved, but mild diplopia remained.

## Discussion

Hypernatremia is defined as a serum sodium concentration above 145 mEq/L, and it is most often caused by water loss from the gastrointestinal tract due to vomiting or diarrhea, water loss from the skin by sweating, or loss in the urine due to diabetes insipidus, administration of diuretics, recovery from renal failure, or glucosuria. Less commonly, hypernatremia results from the administration of salt in excess of water, such as after intravenous administration of hypertonic saline and salt tablets [8]. In addition, it may occur with the use of certain drugs, such as lithium and valproate [9,10], and it has also been shown to occur in patients treated with excess sodium bicarbonate to correct metabolic acidosis [11]. In this case, acute hypernatremia resulted from intravenous sodium bicarbonate therapy for the treatment of severe metabolic acidosis and altered mental status in a patient with symptomatic distal RTA and Sjögren’s syndrome.

Osmotic disturbances like rapid correction of hyponatremia can be associated with demyelination of the pons, causing CPM. The disruption of blood brain barrier (BBB) occur secondary to osmotic stress and is thought to be one of the leading factors in pathogenesis of CPM. In fact, in a murine experiment, BBB disruption during the first 24 hours of hyponatremia was associated with a 70% risk of demyelination [12]. CPM can occur in the setting of several osmotic challenges. First, rapid and excessive correction of sodium in hyponatremia, usually an increase in sodium concentration of more than 18 mg/dL in the first 48 hours, can be associated with CPM. Second, in patients with chronic liver disease and alcoholism, CPM can be associated with acute hypernatremia and hyperglycemia after administration of lactulose for the treatment of hepatic encephalopathy [13]. Third, CPM is associated with acute hypernatremia [3,4] and the rapid correction of hypernatremia [2,4]. The relationship between acute hypernatremia and CPM is well characterized in animal experiments, showing acute hypernatremia to result in brain myelinolysis and cellular necrosis [14]. Additionally, animal experiments demonstrated that the osmotic gradient necessary to induce brain myelinolysis is higher for normonatremic than for hyponatremic rats [15]. In our case, an overdose of intravenous sodium bicarbonate was associated with not only acute hypernatremia, but also CPM.

Because there are no proven effective therapies for CPM, its prevention is essential. Supportive therapy should be provided to all patients who were functional prior to the onset of CPM and should be continued for at least six to eight weeks before drawing conclusions about the severity of the deficits and their irreversibility [16]. There have been some animal studies investigating the benefits of re-inducing hyponatremia in the case of rapid overcorrection of hyponatremia in order to avoid osmotic demyelination. Gankam Kengne et al. demonstrated that re-induction of hyponatremia by an intraperitoneal administration of water 12 hours after rapid overcorrection of hyponatremia effectively prevented the opening of the BBB, reduced neurological manifestations, decreased microglial activation, and resulted in a significant decrease in mortality in rats [17]. The rat experiment by Soupart et al. showed that after exposure to an excessive correction of chronic hyponatremia, even when rats have developed myelinolysis-related neurologic symptoms, hypotonic fluids administration could improve survival and could prevent the subsequent development of brain myelinolysis [18].

Although there are no well-studied therapies for CPM, plasma exchange might be a beneficial therapy when CPM is associated with rapid correction of hyponatremia. For example, according to Bibl et al., three young female patients with CPM were successfully treated with extensive therapeutic plasma exchange [5]. These three patients were treated with plasma exchange for three to seven weeks, and their neurological symptoms were improved within two to twelve months. Because undefined myelin-toxic compounds released by osmotic stress contribute to the irreversible demyelinating process in CPM, therapeutic plasma exchange may exert its effect by reducing these high-molecular weight myelin-toxic substances, leading to clinical improvement [5]. This theory may explain why plasma exchange was effective in our case of CPM due to acute hypernatremia. Our patient’s neurological symptoms, which included dysarthria, difficulty swallowing, and tetraparesis, were markedly improved the day after the two-session regimen of plasma exchange. However, mild diplopia did remain after one year.

Our patient’s course supports the concept that intravenous sodium bicarbonate therapy for the treatment of metabolic acidosis can be associated with acute hypernatremia and CPM. In the setting of acute hypernatremia, CPM may be successfully treated with plasma exchange, likely due to the rapid correction of the hyponatremia.

## Conclusion

Our case indicates that serum sodium concentrations should be carefully monitored in patients with distal RTA who are receiving intravenous sodium bicarbonate therapy. We should keep in mind that acute hypernatremia and CPM can be associated with intravenous sodium bicarbonate therapy, and CPM due to acute hypernatremia may be effectively treated with plasma exchange.

## Consent

Written informed consent was obtained from the patient for publication of this Case report and any accompanying images. A copy of the written consent is available for review by the Editor of this journal.

## Abbreviations

ODS: Osmotic demyelination syndrome; CPM: Central pontine myelinolysis; EPM: Extra-pontine myelinolysis; RTA: Renal tubular acidosis; TTKG: Transtubular potassium gradient; BBB: Blood brain barrier.

## Competing interests

The authors declare that they have no competing interests.

## Authors’ contributions

KYC, IHL, GJK, KWC, HSP and HWK treated the patient and provided data about the history and laboratory results in this report. The manuscript was prepared by KYC and HWK. All authors participated in discussions about the manuscript and approved the final version.

## Pre-publication history

The pre-publication history for this paper can be accessed here:

http://www.biomedcentral.com/1471-2369/15/56/prepub
